# Diagnostic Challenge and Treatment Delay in Drowning‐Associated Pneumonia: A Case of Combined *Aeromonas*, *Legionella*, and *Aspergillus* Infection

**DOI:** 10.1155/crdi/8851440

**Published:** 2026-02-13

**Authors:** Takafumi Masuda, Kazuki Tanaka, Akira Kawamura, Ryo Suzuki, Takumi Nagasaki, Kotaro Yamada, Ryuuichi Nakamura, Toshiya Hiramatsu, Kana Uchida, Norimichi Akiyama, Shun Matsuura, Naoki Koshimizu

**Affiliations:** ^1^ Department of Respiratory Internal Medicine, Fujieda Municipal General Hospital, Fujieda, 426-8677, Shizuoka, Japan, hospital.fujieda.shizuoka.jp; ^2^ Department of Emergency Medicine, Fujieda Municipal General Hospital, Fujieda, 426-8677, Shizuoka, Japan, hospital.fujieda.shizuoka.jp

**Keywords:** *Aeromonas hydrophila*, *Aspergillus fumigatus*, bronchoscopy, case report, combined infection, drowning-associated pneumonia

## Abstract

Drowning‐associated pneumonia often involves multiple types of pathogens, including *Aeromonas hydrophila, Legionella* spp., and fungi. We report the case of an 82‐year‐old man who developed a rare combined infection with these three organisms after freshwater drowning. Initial therapy for typical aspiration pneumonia was ineffective. Although *A. hydrophila* and *Legionella pneumophila* were subsequently identified and targeted, the patient’s condition failed to improve. While chest computed tomography on the 11th day of hospitalization revealed worsening infiltrates and new cavity lesions, bronchoscopy was delayed until Day 13 due to circulatory instability. Subsequently, *Aspergillus fumigatus* was identified from bronchial lavage. Despite starting targeted antimicrobial and antifungal therapy late, the patient died on Day 21. This case highlights that in freshwater drowning, immediate broad‐spectrum empirical treatment covering high‐risk environmental bacteria and *Legionella* is essential. Furthermore, if initial therapy is ineffective or radiological progression occurs, early bronchoscopy must be performed to identify secondary or opportunistic pathogens, including *Aspergillus* or other fungi to prevent deadly delays in diagnosis.

## 1. Introduction

Pneumonia is a common and serious complication of drowning, and the causative pathogens often vary depending on the water environment. Although initial treatment typically targets aspiration pneumonia pathogens, drowning‐associated pneumonia (DAP) poses major diagnostic challenges because infections are frequently polymicrobial and may involve environmental bacteria and fungi that are resistant to standard empirical antibiotics [1].

In addition, the clinical course is often affected by a “diagnostic lag,” in which secondary or opportunistic pathogens, such as *Aspergillus* spp. or other fungi, are not recognized until the patient fails to respond to initial therapy [2]. Delays in identifying these pathogens and initiating appropriate treatment are associated with poor clinical outcomes.

Among the various causative organisms, *Aeromonas hydrophila* is a well‐recognized and highly virulent pathogen in freshwater drowning and can cause severe drowning‐related pneumonia [3, 4]. Therefore, it should be strongly considered when a patient presents with pneumonia after drowning. Additionally, *Aspergillus* and *Scedosporium* spp. are also known causes of severe DAP [5–7].

We hereby report a case of a patient suffering from DAP who was infected by a combination of *A. hydrophila*, *Legionella*, and *Aspergillus fumigatus*.

## 2. Case Presentation

An 82‐year‐old man was transported to our institution after a bicycle accident in which he collided with a car and fell into an irrigation canal approximately 90 cm wide and 90 cm deep. The canal water was stagnant and heavily contaminated with mud and soil due to the active agricultural season. His medical history included Type 2 diabetes, prior acute myocardial infarction, and dyslipidemia, and he had a 10‐year smoking history.

Upon arrival, the patient was alert but exhibited retrograde amnesia and repeated vomiting, consistent with a concussion. His vital signs were stable (temperature: 36.3°C), though he required 3 L/min of oxygen via nasal cannula. Chest auscultation revealed bilateral coarse crackles. Laboratory tests showed no evidence of systemic inflammation (white blood cell count: 7.8 × 10^9^/L [normal range: 3.3–8.6 × 10^9^/L]) and C‐reactive protein (CRP) level: 0.01 mg/dL (normal range: 0.01–0.14 mg/dL), and liver and kidney function tests were within normal limits.

**FIGURE 1:**
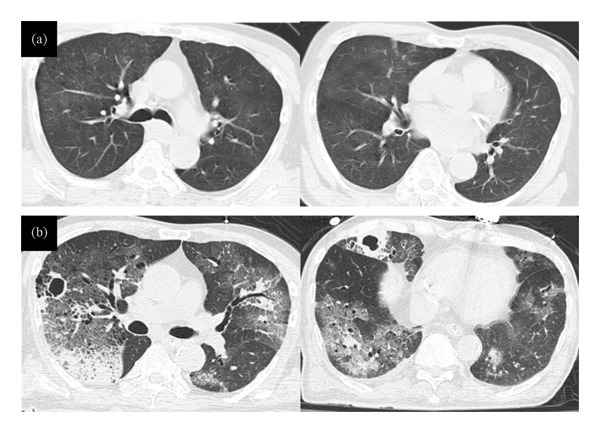
Changes in the imaging findings on chest CT scan. On admission, widespread pale ground‐glass opacities were observed in both lungs due to water aspiration (a). Ground‐glass opacity and infiltrating shadows were identified mainly around the bilateral airways on Day 11. New hollow shadows were also noted in the right upper and middle lobe on Day 11 (b). Left: images of the upper lobes; right: images of the middle and lower lobes.

Although the chest radiograph showed no abnormalities, chest computed tomography (CT) revealed bilateral ground‐glass opacities (Figure 1(a)). Detailed review of the CT scan showed only chronic emphysematous changes consistent with his smoking history, with no evidence of focal consolidation or active inflammatory lesions. Pre‐existing pneumonia was therefore ruled out based on these findings and the absence of preceding respiratory symptoms. Furthermore, a nasogastric tube inserted for persistent vomiting revealed gravel mixed with gastric aspirate. These findings—concussion, vomiting, and the presence of gravel in the stomach—suggested that airway protection was transiently compromised, leading to aspiration of a significant volume of contaminated water.

The patient was admitted for observation. During the first night, his condition worsened, with a fever of 38°C and increasing respiratory distress requiring 5 L/min of oxygen. A diagnosis of acute aspiration pneumonia was made, and treatment with ampicillin/sulbactam (3 g every 8 h for 4 days) was initiated.

**FIGURE 2:**
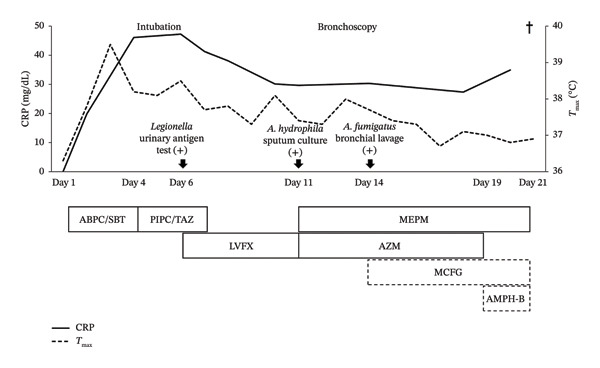
Clinical course and treatment drugs. The solid squares represent the antibiotics administered. The dotted squares represent the antifungal agents administered. The abbreviations and administered doses were as follows: CRP: C‐reactive protein; *T*_max_: maximum temperature, ABPC/SBT: ampicillin/sulbactam (3 g every 8 h); PIPC/TAZ: piperacillin/tazobactam (4.5 g every 8 h); MEPM: meropenem (1 g every 12 h); LVFX: levofloxacin (500 mg on the first day, followed by 250 mg every 24 h); AZM: azithromycin (500 mg every 24 h); MCFG: micafungin (300 mg every 24 h); AMPH‐B: Amphotericin‐B (4 mg/kg every 24 h).

On Hospital Day 4, the antibiotic regimen was changed to piperacillin/tazobactam (4.5 g every 8 h for 4 days) due to a marked elevation in serum CRP levels (46.1 mg/dL) and worsening pulmonary infiltrates (Figure 2). However, his respiratory status remained unstable with profuse sputum production, and he required intubation and mechanical ventilation on Day 5. After a urinary antigen test returned positive for *Legionella*, levofloxacin was added on Day 6 (500 mg loading dose, followed by 250 mg daily for 5 days, adjusted for renal function) (Figure 2).

Despite these interventions, the patient remained febrile (approximately 38 °C) with persistently high inflammatory markers (CRP: 30–40 mg/dL). On Day 11, repeat CT scan revealed scattered ground‐glass opacities and consolidation, along with new cavity lesions in the right lobes (Figure 1(b)). Sputum cultures from the same day yielded *A. hydrophila* and quinolone‐resistant *Escherichia coli*. Therefore, respiratory specialists diagnosed refractory polymicrobial infection acquired during freshwater drowning. The presence of cavitary lesions raised concern for necrotizing pneumonia or concomitant fungal infection, such as aspergillosis.

Consequently, the treatment regimen was escalated to meropenem (1 g every 12 h for 11 days, adjusted for renal function) and azithromycin (500 mg once daily for 8 days) to cover carbapenem‐sensitive *Aeromonas* and *Legionella* (Figure 2). Although urgent bronchoscopy was planned to obtain a definitive diagnosis, the procedure was postponed until Day 13 because of circulatory instability requiring vasopressor support.

**FIGURE 3:**
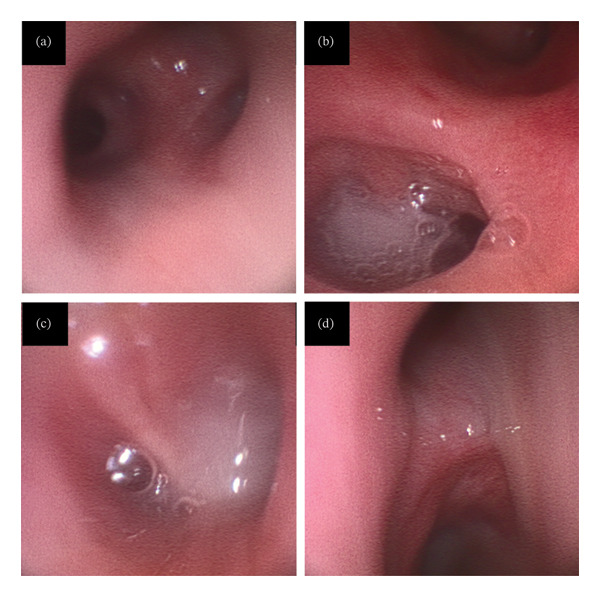
Bronchial lumen on bronchoscopy on Day 13. A large amount of white sputum was noted adhering to the bronchial wall. Bronchial lavage revealed the presence of *Aspergillus fumigatus* on the right upper (a), middle (b), and lower (c) lobe bronchus and left lower one (d).

Bronchoscopy performed on Day 13 revealed extensive white mucous plaques adhering to the bronchial walls, particularly in the right lung (Figure 3). Bronchial lavage from the cavitary areas yielded *A. fumigatus* from multiple lobes. On Day 14, the patient’s level of consciousness declined; given concern for possible central nervous system involvement from invasive aspergillosis, micafungin (300 mg once daily for 7 days) was initiated (Figure 2). Despite the subsequent addition of Amphotericin B (4 mg/kg once daily for 3 days), his condition continued to deteriorate, and the patient succumbed on Day 21.

**FIGURE 4:**
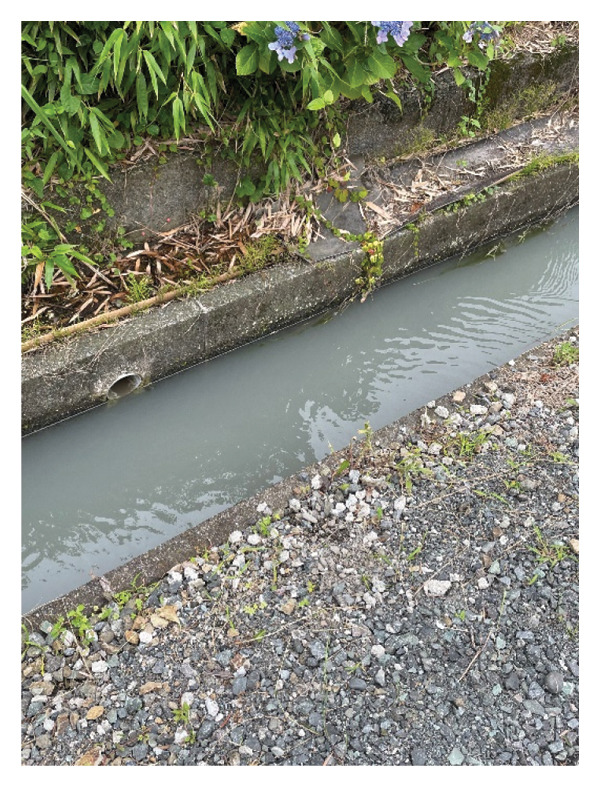
Image of the canal at the scene of the traffic accident. Water was flowing in from nearby rice fields. In addition to *Aeromonas hydrophila*, a diverse range of microorganisms was identified in the water sample (see Table 1).

Subsequent environmental analysis confirmed the canal water as the likely source of infection (Figure 4). Water samples were collected in a sterile container that had been prerinsed with site water, leaving headspace for aeration. The specimens were promptly transported to the laboratory and cultured on standard media (blood agar and MacConkey agar) at 35°C. This procedure led to the identification of *A. hydrophila*, matching the strain isolated from the patient’s sputum. Furthermore, as detailed in Table 1, the environmental analysis revealed a diverse range of microorganisms at the scene, reflecting the heavily contaminated nature of the canal and the high potential for polymicrobial aspiration.

**TABLE 1:** Microorganisms identified from the environmental water sample collected at the scene.

Category	Species identified
Genus *Aeromonas*	*A. hydrophila*, *A. veronii*
Environmental bacteria	*Pseudomonas fluorescens/putida*, *Roseomonas* sp., *Brevundimonas diminuta*, *Chromobacterium violaceum*
Enterobacteriaceae	*Enterobacter cloacae*, *E. intermedium*, *Cronobacter sakazakii*, *Klebsiella oxytoca*, *K. ozaenae*

## 3. Discussion

We have hereby reported a case of combined infection caused by *A. hydrophila*, *Legionella* spp., and *A. fumigatus* in an 82‐year‐old male after drowning. In this case, the patient was unresponsive to antibiotic therapy, despite detailed examination that included frequent sputum cultures and bronchoscopy.

Pneumonia is the most common and serious complication of drowning, with a prevalence of 24%–51%, and a mortality rate of 18%–81% [1]. Notably, its causative pathogens can vary based on the nature of the aspirated water. A systematic review and meta‐analysis conducted by Cousin VL *et al.* reported that *Aeromonas* spp. were the most common pathogen detected, followed by *Haemophilus influenzae* in 106 cases of pneumonia that developed after drowning in fresh water [1]. These observations align with the course of this case, in which the patient drowned in fresh water. Furthermore, several *Aeromonas* spp. are resistant to ampicillin. Results of a study conducted by Honda et al. involving 18 cases of *A. hydrophila*‐related pneumonia demonstrated that the rates of resistance to penicillin and first‐generation cephems, quinolone, and carbapenem were 100%, 11%, and 11%, respectively. However, no resistance was detected to both quinolone as well as carbapenem [3]. *A. hydrophila,* the organism that was identified in the sputum culture in this case was resistant to ampicillin, ampicillin/sulbactam, and cefazolin. Similar to previous reports, *A*. *hydrophila*‐related pneumonia after drowning is highly resistant to ampicillin.

Reports on fungal infection associated with drowning are scarce. One study reported that aspiration pulmonary aspergillosis can be caused by multiple species of fungi including *A. fumigatus*, after drowning in rice paddies and other places [5]. *A*. *fumigatus* has also been reported to invade the central nervous system and cause neurological disorders. The risk factors include concomitant aspiration pneumonia, warm weather and seasons, invasive pulmonary aspergillosis, and delays in diagnosis and administration of antifungal medications [8]. In the current case, the risk factors include the timing of the accident and the concomitant occurrence of aspiration pneumonia.

In addition to *Aspergillus* species, *Scedosporium* species (e.g., *S. apiospermum complex*) are recognized as important and highly virulent fungal pathogens after freshwater drowning. These fungi are ubiquitous in soil and stagnant water, and their clinical significance was notably highlighted after the Great East Japan Earthquake, when many cases of “tsunami lung” were associated with *Scedosporium* infections [9, 10]. Unlike many other fungi, *Scedosporium* is often resistant to Amphotericin B and may disseminate to the central nervous system, leading to high mortality rates [11]. Because fungal cultures are time‐consuming and diagnosis is often delayed, several studies recommend early empirical antifungal therapy—particularly with voriconazole, which is active against both *Aspergillus* and *Scedosporium*—in patients who develop severe respiratory failure or do not respond to initial broad‐spectrum antibiotics after drowning [8, 9]. This case further underscores the need to consider a broad range of environmental fungi from the outset.

In this case, multiple bacterial species observed in the cultures made it challenging to estimate the pathogen and select the appropriate treatment strategy. The water in the canal at the site of the traffic accident was confirmed to be flowing from a nearby rice paddy field. The water sample was submitted for culturing and *A. hydrophila* was detected, but no traces of *A. fumigatus* and *Legionella* spp. were found. The number of bacteria in the environmental water could have fluctuated considering the number of days that had passed since the traffic accident occurred, which could be the reason why *A. fumigatus* and *Legionella* spp. were not detected.

Early identification of multiple pathogens in DAP is challenging but crucial. As shown in Table 1, the environmental water contained a diverse range of Gram‐negative rods, including *Pseudomonas* and Enterobacteriaceae species. Although not all were isolated from the patient, their presence highlights the heavily contaminated nature of the site. Clinicians should maintain a high index of suspicion for polymicrobial infection based on the following indicators:

1. Environmental and Exposure History: Drowning in freshwater sources rich in organic matter, such as rice paddies, stagnant ponds, or floodwaters, significantly increases the risk of inhaling a diverse microbial flora, including *Aeromonas* spp., *Legionella* spp., and soil‐borne fungi.
2. Radiological Patterns and Progression: Initial CT scans may show nonspecific ground‐glass opacities; however, rapid progression to dense consolidation or the early appearance of cavitary lesions—especially in immunocompromised or older adult patients—strongly suggests involvement of aggressive environmental bacteria or opportunistic fungi such as *Aspergillus* and other fungi.
3. Treatment Refractoriness: Failure to respond to standard empirical therapy for aspiration pneumonia (e.g., ampicillin/sulbactam) within the first 48–72 h is a key diagnostic clue. Persistent high fever and rising inflammatory markers (e.g., CRP) despite beta‐lactam therapy should prompt escalation to broader‐spectrum antibiotics and further diagnostics such as bronchoscopy.

By integrating these environmental, radiological, and clinical indicators, clinicians can more accurately determine when to shift from narrow‐spectrum to broad‐spectrum empirical therapy to cover multiple resistant organisms.

Proposed strategies for reducing diagnostic delays to improve the clinical course of DAP included several proactive measures as follows. First, early bronchoscopy and bronchial lavage are essential. In this case, bronchoscopy was delayed until Day 13 due to the patient’s circulatory instability. However, relying solely on sputum cultures can lead to a “diagnostic lag,” as environmental pathogens and fungi are often not detected in expectorated samples. Early bronchial lavage allows direct sampling of the lower respiratory tract, facilitating rapid identification of pathogens through culture and potential PCR‐based assays. Second, careful selection of initial empirical antimicrobial therapy is critical for a prognosis. Although ampicillin/sulbactam is a standard treatment for typical aspiration pneumonia, recent meta‐analysis indicates that approximately 30% of pathogens isolated in DAP are resistant to amoxicillin–clavulanate. In freshwater drowning, *A. hydrophila* is a major pathogen with an 18% prevalence, which frequently produces inducible chromosomal beta‐lactamases, rendering standard aminopenicillin/beta‐lactamase inhibitor combinations inadequate. Given the rapid progression of such infections, broad‐spectrum therapy should be initiated immediately upon admission. Based on the microbiological ecology of DAP, piperacillin–tazobactam and fourth‐generation cephalosporins (e.g., cefepime) are recommended as suitable empirical agents [1]. Furthermore, since these regimens may not cover *Legionella* species—another critical pathogen in freshwater—the inclusion of a respiratory fluoroquinolone (e.g., levofloxacin) is highly effective, providing comprehensive coverage for both *Aeromonas* spp. and *Legionella* spp. Adopting such a proactive approach, rather than waiting for culture results, is essential to prevent rapid clinical deterioration. Third, a low threshold for initiating empirical antifungal therapy is warranted. Drowning in soil‐contaminated water (e.g., rice paddies) carries a high risk of *Aspergillus* or *Scedosporium* infection. Rather than awaiting culture confirmation, the appearance of cavitary lesions on CT or failure to improve inflammatory markers within the first week should prompt the addition of mold‐active antifungals such as voriconazole. Implementing this proactive rather than reactive strategy may significantly reduce mortality in complex polymicrobial drowning‐associated infections.

In conclusion, DAP frequently involves high‐virulence pathogens and fungi that are resistant to standard treatments for aspiration pneumonia. To improve patient outcomes, clinicians should suspect polymicrobial infection when environmental risk factors are present (e.g., drowning in stagnant freshwater), radiological findings show rapid progression (e.g., early cavitation), or there is clinical failure to respond to standard beta‐lactams therapy. In such cases, immediate empirical therapy covering both environmental bacteria (e.g., *Aeromonas* spp.) and *Legionella* spp.—using agents such as fourth‐generation cephalosporins or carbapenems combined with fluoroquinolones—is essential. Furthermore, early diagnostic bronchoscopy is critical to identify secondary or opportunistic pathogens, including *Aspergillus* spp. and other fungi, allowing for timely initiation of targeted therapy.

**Nomenclature**
DAP: Drowning‐associated pneumonia
CRP: C‐reactive protein
CT: Computed tomography

## Author Contributions

Drafting of the text, sourcing and editing of clinical images, investigation results, and critical revision for important intellectual content: Takafumi Masuda, Kazuki Tanaka, Akira Kawamura, Ryo Suzuki, Takumi Nagasaki, Kotaro Yamada, Ryuuichi Nakamura, Toshiya Hiramatsu, Kana Uchida, Norimichi Akiyama, Shun Matsuura, and Naoki Koshimizu. Final approval of the manuscript: Kazuki Tanaka, Akira Kawamura, Ryo Suzuki, Takumi Nagasaki, Kotaro Yamada, Ryuuichi Nakamura, Toshiya Hiramatsu, Kana Uchida, Norimichi Akiyama, Shun Matsuura, and Naoki Koshimizu.

## Funding

The authors received no specific funding for this work.

## Consent

Written informed consent was obtained from the patient’s son for the publication of this manuscript and any accompanying images.

## Conflicts of Interest

The authors declare no conflicts of interest.

## Supporting Information

File S1: CARE Checklist. The completed CARE guidelines checklist for this case report is provided as Supporting Information.

## Supporting information


**Supporting Information** Additional supporting information can be found online in the Supporting Information section.

## Data Availability

Data sharing is not applicable to this article as no new data were created or analyzed in this study.

## References

[1] Cousin V. L. and Pittet L. F. , Microbiological Features of Drowning-Associated Pneumonia: a Systematic Review and Meta-Analysis, Annals of Intensive Care. (2024) 14, no. 1, 10.1186/s13613-024-01287-1. PMC11031557 38641650

[2] Koide S. , Hadano Y. , Mizuochi S. , Koga H. , and Yamashita H. , Invasive Aspergillosis After Non-fatal Drowning, International Medical Case Reports Journal. (2020) 13, 77–83, 10.2147/IMCRJ.S241234. 32210640 PMC7069574

[3] Honda M. , Okumura H. , Inoue T. , and Maekawa T. , A Case of Surviving the Fulminant Form of Aeromonas hydrophila Pneumonia due to Aspiration of River Water, JJAAM. (2014) 25, 717–722, 10.3893/jjaam.25.717.

[4] Tadié J. M. , Heming N. , Serve E. et al., Drowning Associated Pneumonia: a Descriptive Cohort, Resuscitation. (2012) 83, 399–401, 10.1016/j.resuscitation.2011.08.023, 2-s2.0-84857042606. 21907690

[5] Yamawaki S. , Nakashima K. , Suzuki F. et al., Rice-Field Drowning-Associated Pneumonia in Which Pseudomonas spp., Aspergillus fumigatus, and Cunninghamella Sp. Are Isolated, Internal Medicine. (2016) 55, no. 7, 825–829, 10.2169/internalmedicine.55.4454, 2-s2.0-84962174322. 27041173

[6] Jenks J. D. and Preziosi M. , A Challenging Case of Invasive Pulmonary Aspergillosis After Near-Drowning: a Case Report and Literature Review, Infectious Diseases in Clinical Practice. (2015) 23, no. 5, 227–230, 10.1097/IPC.0000000000000263, 2-s2.0-84941282201. 26392737 PMC4574302

[7] Peng Y. , Chen J. , Wang H. , Jia Q. , Xie J. , and Mo G. , A Systemic Infection Involved in Lung, Brain and Spine Caused by Scedosporium Apiospermum Species Complex After Near-Drowning: a Case Report and Literature Review, BMC Infectious Diseases. (2024) 24, 10.1186/s12879-023-08279-9. PMC10956195 38515075

[8] Leroy P. , Smismans A. , and Seute T. , Invasive Pulmonary and Central Nervous System Aspergillosis After Near-Drowning of a Child: Case Report and Review of the Literature, Pediatrics. (2006) 118, no. 2, e509–e513, 10.1542/peds.2005-2901, 2-s2.0-33748434377. 16864641

[9] Nakamura Y. , Utsumi Y. , Suzuki N. et al., Multiple Scedosporium Apiospermum Abscesses in a Woman Survivor of a Tsunami in Northeastern Japan: a Case Report, Journal of Medical Case Reports. (2011) 5, no. 1, 10.1186/1752-1947-5-526, 2-s2.0-80054856702. PMC3223506 22027347

[10] Nakadate T. , Nakamura Y. , Yamauchii K. , and Endo S. , Two Cases of Severe Pneumonia After the 2011 Great East Japan Earthquake, Western Pacific Surveill Response Journal. (2012) 3, no. 4, 67–70, 10.5365/WPSAR.2012.3.2.002. PMC3729095 23908944

[11] Neoh C. F. , Chen S. C.-A. , Lanternier F. et al., Scedosporiosis and Lomentosporiosis: Modern Perspectives on These difficult-to-treat Rare Mold Infections, Clinical Microbiology Reviews. (2024) 37, no. 2, 10.1128/cmr.00004-23. PMC11237582 38551323

